# Unraveling Massive Crocins Transport and Accumulation through Proteome and Microscopy Tools during the Development of Saffron Stigma

**DOI:** 10.3390/ijms18010076

**Published:** 2017-01-01

**Authors:** Lourdes Gómez-Gómez, Verónica Parra-Vega, Alba Rivas-Sendra, Jose M. Seguí-Simarro, Rosa Victoria Molina, Claudia Pallotti, Ángela Rubio-Moraga, Gianfranco Diretto, Alicia Prieto, Oussama Ahrazem

**Affiliations:** 1 Botanical Institute, Department of Science Technology, Agroforestry and Genetics, Faculty of Pharmacy, University of Castilla-La Mancha, Campus Universitario s/n, 02071 Albacete, Spain; marialourdes.gomez@uclm.es (L.G.-G.); angela.rubio@uclm.es (A.R.-M.); 2 Cell Biology Group, COMAV Institute, Polytechnic University of Valencia, 46071 Valencia, Spain; veparve@upv.es (V.P.-V.); alriis@upvnet.upv.es (A.R.-S.); seguisim@btc.upv.es (J.M.S.-S.); 3 Department of Vegetal Biology, Polytechnic University of Valencia, 46071 Valencia, Spain; rvmolina@bvg.upv.es (R.V.M.); pallotti@bvg.upv.es (C.P.); 4 Italian National Agency for New Technologies, Energy, and Sustainable Development, Casaccia Research Centre, 00123 Rome, Italy; gianfranco.diretto@enea.it; 5 The Biological Research Center (CIB) Spanish National Research Council (CSIC), C/Ramiro de Maeztu 9, 28040 Madrid, Spain; aliprieto@cib.csic.es; 6 Faculty of Environmental Sciences and Biochemistry Toledo, University of Castilla-La Mancha, Campus Tecnológico de la Fábrica de Armas, Avda, Carlos III, s/n, 45071 Toledo, Spain

**Keywords:** chromoplast, crocetin, crocins, proteome, transport

## Abstract

Crocins, the glucosides of crocetin, are present at high concentrations in saffron stigmas and accumulate in the vacuole. However, the biogenesis of the saffron chromoplast, the changes during the development of the stigma and the transport of crocins to the vacuole, are processes that remain poorly understood. We studied the process of chromoplast differentiation in saffron throughout stigma development by means of transmission electron microscopy. Our results provided an overview of a massive transport of crocins to the vacuole in the later developmental stages, when electron dense drops of a much greater size than plastoglobules (here defined “crocinoplast”) were observed in the chromoplast, connected to the vacuole with a subsequent transfer of these large globules inside the vacuole. A proteome analysis of chromoplasts from saffron stigma allowed the identification of several well-known plastid proteins and new candidates involved in crocetin metabolism. Furthermore, expressions throughout five developmental stages of candidate genes responsible for carotenoid and apocarotenoid biogenesis, crocins transport to the vacuole and starch metabolism were analyzed. Correlation matrices and networks were exploited to identify a series of transcripts highly associated to crocetin (such as 1-Deoxy-d-xylulose 5-phosphate synthase (*DXS*), 1-Deoxy-d-xylulose 5-phosphate reductoisomerase (*DXR*), carotenoid isomerase (*CRTISO*), Crocetin glucosyltransferase 2 (*UGT2*), etc.) and crocin (e.g., ζ-carotene desaturase (*ZDS*) and plastid-lipid-associated proteins (*PLAP2*)) accumulation; in addition, candidate aldehyde dehydrogenase (*ADH*) genes were highlighted.

## 1. Introduction

Saffron spice is produced from the dried stigmas of *Crocus sativus* L., these long scarlet stigmas of saffron are highly valued for flavoring and coloring foods and are among the most expensive spices in the world. The compounds responsible for the organoleptic properties of the spice are the following apocarotenoids: crocins, the glucosylated derivatives of the apocarotenoid crocetin, which gives stigma its red coloration, and picrocrocin, a monoglucosylated derivative of 4-hydroxy-2,6,6-trimethyl-1-cyclohexene-1-carboxaldehyde (HTCC), which provides saffron its bitter taste, while its degradation generates safranal, responsible for the aroma [1]. Saffron has been used in traditional medicine and has been reported to have a variety of health benefits [2]. Among these properties, saffron has been used as analgesic and sedative and shows growth inhibition against certain lines of malignant cells, suggesting that saffron might be used as an anticancer agent [3]. These properties are due to the apocarotenoids crocetin, crocins and picrocrocin, the main components of the stigma and spice, which are also present in other Crocus species, although in minor quantities [4,5]. Zeaxanthin, synthesized in the saffron chromoplast through the carotenoid biosynthetic pathway [4,6], is the precursor of these saffron apocarotenoids [7]. The enzyme carotenoid cleavage dioxygenase 2 (CCD2), localized in the chromoplast [8], catalyzed the cleavage of the 7,8 and 7′,8′ double bonds of this carotenoid to render one molecule of crocetin dialdehyde and two molecules of 8-hydroxy-β-cyclocitral [9]. These products are further oxidated and glucosylated [10], generating soluble apocarotenoids which accumulate in the vacuole [11].

Chromoplasts are present in many flowers and fruits giving them the yellow, orange and red colors [12]. They are also found in some roots, and tubers such as carrots, and sweet potatoes [13], respectively. In saffron, reticulotubular structures in the chromoplast made up of a reticulum of tubules are abundant, but vesicle and globule forms also coexist [11].

The process of crocins transport from the chromoplast to the vacuole is not fully understood. To address this question, we monitored chromoplast development during stigma growth by means of transmission electron microscopy. Furthermore, we checked the expression of the putative genes responsible for chromoplast biogenesis, crocetin formation and crocins transport to the vacuole and we used a series of bioinformatics approaches, mainly based on correlation analysis, to evidence transcripts potentially involved in apocarotenoid synthesis, movement and storage. Finally, we analyzed the chromoplast proteome from the stigma at the red-scarlet stage by through nanoscale liquid chromatography coupled to tandem mass spectrometry (nanoLC-MS/MS) in order to determine the potential players involved in the crocins transport. Overall, our data demonstrated that a mechanism involving mass transport of crocins from crocinoplast to the central vacuole exists in saffron stigmas. This process consists in an increased crocins concentration that triggers their packaging in vesicles, which develop and ultimately merge with the vacuole to deliver crocins inside.

## 2. Results

### 2.1. Ultrastructural Characterization of Chromoplast Biogenesis in Saffron

**Figure 1:**
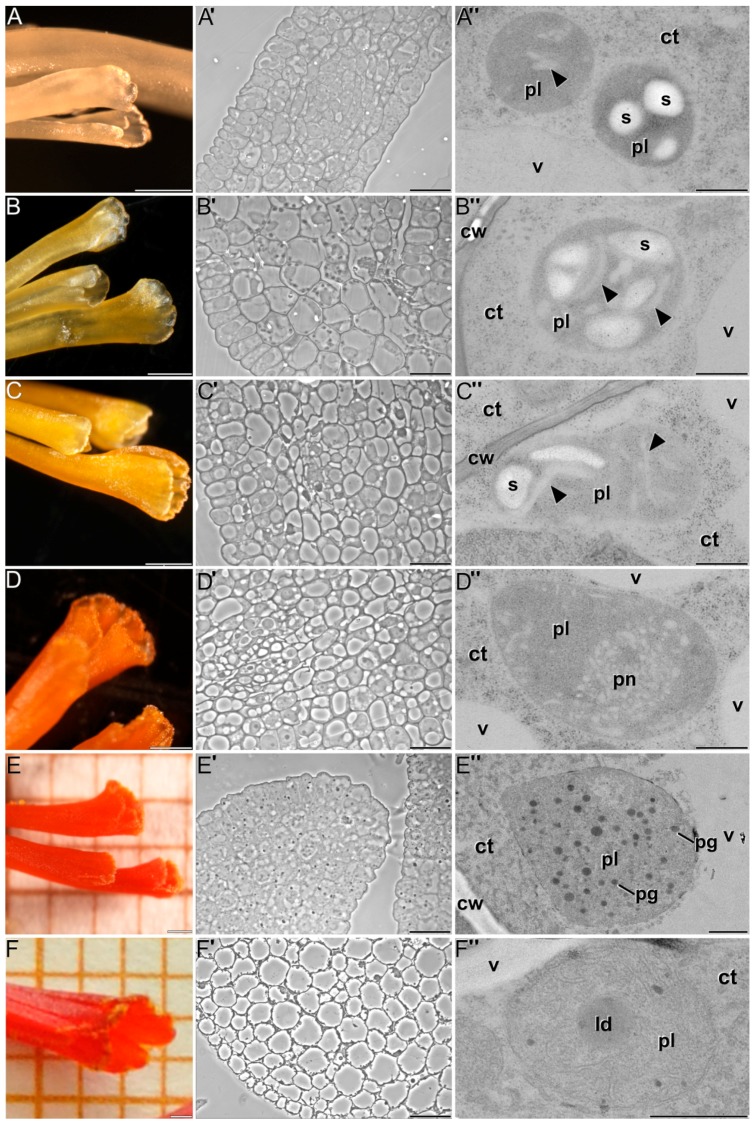
The different stages of saffron stigma development: white (**A**–**A″**); yellow (**B**–**B″**); orange-yellow (**C**–**C″**); orange (**D**–**D″**); red (**E**–**E″**); and red-scarlet (**F**–**F″**). A general microscopy overview of stigmas at each stage is shown in (**A**–**F**); (**A′**–**F′**) show display light microscope images of tissue organization at each stage; and (**A″**–**F″**) display electron micrographs of plastids at each stage. Arrowheads point to plastid membranous structures. ct: cytoplasm; cw: cell wall; ld: lipid droplet; pg: plastoglobule; pn: polarized network; pl: plastid; s: starch; v: vacuole. Bars in (**A**–**F**): 500 µm; (**A′**–**F′**): 20 µm; (**A″**–**F″**): 500 nm.

Six time-points of fresh stigmas were defined: white (Figure 1A–A″), yellow (Figure 1B–B″), orange-yellow (Figure 1C–C″), orange (Figure 1D–D″), red (Figure 1E–E″) and red-scarlet (Figure 1F–F″), according to [14], were examined by light and transmission electron microscopy. Pistil structural organization was consistent with that previously reported [11]. The stigmas were formed by several layers of parenchymatic cells, covered by a layer of epidermic cells and two vascular bundles run longitudinally the fundamental tissue. During stigma development, this tissue organization was not altered, but remarkable changes were detected at the subcellular level (Figure 1A′–F′).

Overall, cells from earlier developmental stages showed an abundance of organelle-containing cytoplasm, although vacuoles were also present (Figure 1A′–C′). In red stigmas at anthesis, the cells showed a large central vacuole occupying almost all the cell volume (Figure 1F′). In white stigmas, plastids were already detected. They were typically small, round, and included few developing membranous cisternae and/or starch deposits (Figure 1A″). At the yellow stage, plastids increased in size and presented more starch deposits and membranous tubular or cisternal-like elements in the stroma than in the white stage (Figure 1B″). The transition from the yellow to the orange stage was characterized by a reduction in the number and size of starch deposits, while membranous cisternal and tubular elements continued to accumulate (Figure 1C″). At the orange stage, plastids were even larger compared to the previous stages, the starch granules were residual whereas the membranous structures were abundant, forming a polarized network of interconnected tubules and cisternae that occupied an important part of the stroma (Figure 1D″).

Upon reaching the red stage, some plastids still contained few starch deposits, but they were eventually lost at anthesis. From the red stage (Figure 1E″) onwards, the membranous network spanned throughout the entire plastid volume. In addition, plastoglobules of different sizes began to be evident. The membranous network together with the presence of plastoglobules defined the conversion of plastids into chromoplasts, which increased in number and size as red stigmas developed. In addition to these features, we identified the presence of spherical, homogeneously electron dense globules much larger than plastoglobules (Figure 1F″) and not detected in the previous stages.

**Figure 2:**
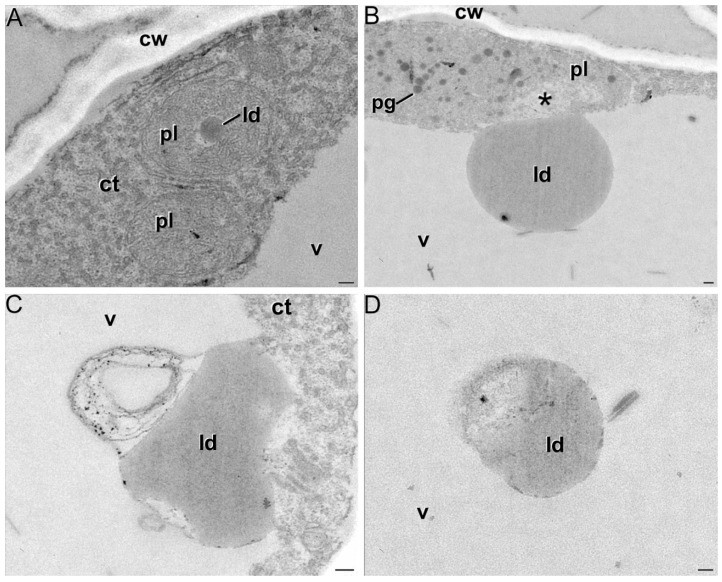
Different stages in lipid droplet transport to the vacuole: (**A**) chromoplast (pl) with a lipid droplet (ld) within; (**B**) droplet being transferred to the vacuole (v) and leaving an empty space (asterisk) in the chromoplast; (**C**) entire chromoplast being degraded while entering the vacuole; and (**D**) lipid droplet being degraded once in the vacuole; ct: cytoplasm; cw: cell wall; pg: plastoglobule. Bars: 100 nm.

**Figure 3:**
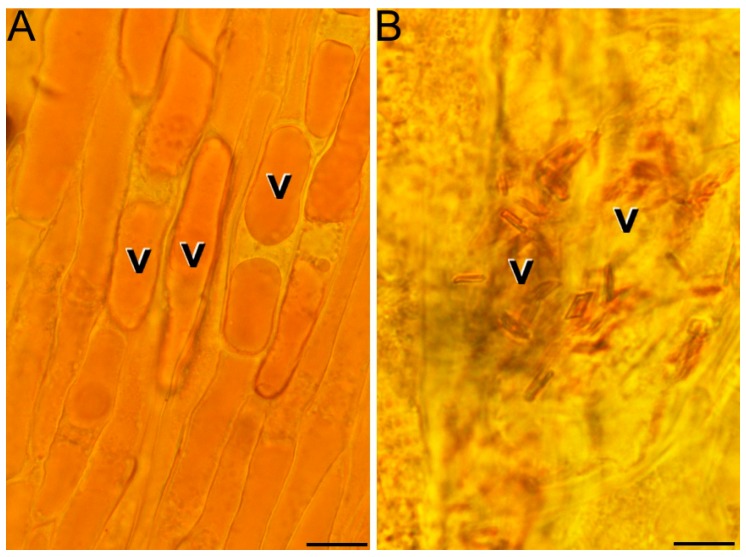
Presence of pigments, both soluble (**A**) and crystalline (**B**), in the vacuole (v) of plasmolyzed cells from red stigmas at anthesis. Bars: 20 µm.

The microscopy studies showed the presence and dynamics of these globules, closely resembling lipid droplets, along the different stages of the red stigma. They continuously increased in size, reaching a maximum diameter of up to 1.5 nm in the late red stigmas. Interestingly, at this stage, the chromoplasts were usually found close to the central vacuole and even connected to it through their membranes (Figure 2A). In such a connection, numerous images suggested a transference of the large globules from the chromoplast to the vacuole (Figure 2B), leaving an empty space in the chromoplast (asterisk in Figure 2B). In some pictures, the whole chromoplast appeared as if it was digested by the vacuole (Figure 2C). In addition, many of these large globules were found within the vacuole and some of them showed signs of rupture and degradation (Figure 2D), evidencing that once into the vacuole, these large lipid droplets started to break down and release their contents to the vacuolar lumen. In addition, we detected in these same samples the presence of red soluble pigments (Figure 3A) and crystals (Figure 3B) in the vacuole when plasmolyzed cells were examined under the light microscope.

### 2.2. Overall Proteomic Analysis

**Figure 4:**
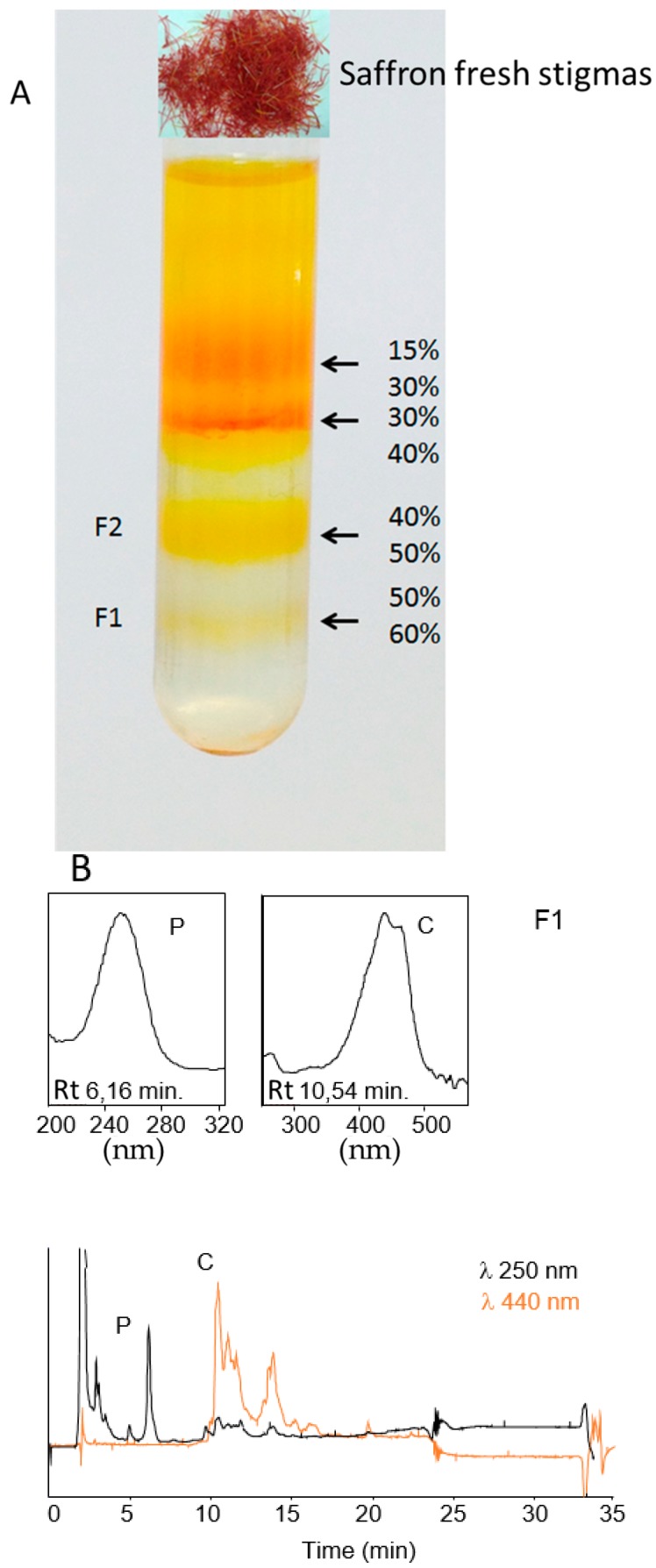
(**A**) Pattern of bands obtained after sucrose density gradient from saffron fresh stigmas; and (**B**) HPLC DAD chromatograms showing the presence of crocins in fraction F1.

**Figure 5:**
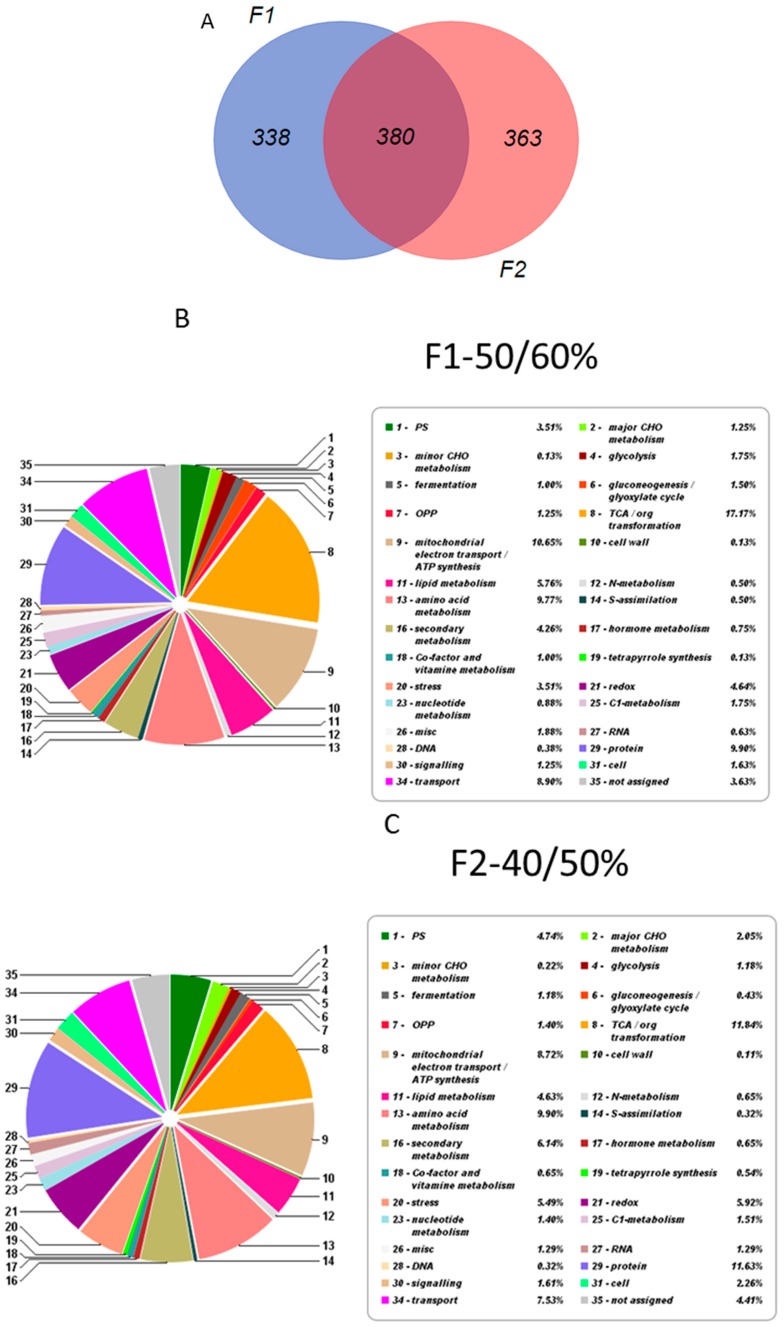
(**A**) Venn diagram obtained after comparison between F1 and F2. Functional category of plastidial proteins identified according to MapMan in: Fraction 1 (**B**); and Fraction 2 (**C**). Functional category of plastidial proteins identified from F1 and F2 fractions according to MapMan.

Four bands (corresponding to the 15%/30%, 30%/40% 40%/50% and 50%/60% interfaces, respectively) were collected and chromoplasts recovered as indicated in the Material and Methods Section (Figure 4). Bands from 15%/30% and 30%/40% have some degraded membranous-like structures when they were checked under microscopy and were not analyzed. High-Performance Liquid Chromatography with Diode-Array Detection (HPLC-DAD) analysis revealed the presence of crocins only in fractions F1 (band from 50%/60%) and F2 (band from 40%/50%) (Figure 4). To further check the purity of the chromoplasts banding at the 40%/50% and 50%/60% interfaces, the activity of specific marker enzymes was determined and related to the total activity measured in the crude cell homogenate. Since succinate dehydrogenase and catalase activity were detected, mitochondrial and peroxisomal contaminations were present in both fractions, although at a low level (less than 5%), while no alcohol dehydrogenase activity was found, thus indicating that the chromoplast preparations were free from cytosolic contamination. The comparison between the two fractions revealed that F1 and F2 shared 380 proteins while 338 and 363 were exclusive to F1 and F2 respectively (Figure 5A). Classification of the identified proteins according to MapMan allowed an overview of the abundance of proteins in various functional classes (Figure 5B,C). The function corresponding to the highest number of proteins was TCA/organic acids transformation in both fractions. Proteins associated with photosynthesis and carbon metabolism, electron transport/ATP synthesis, amino acid metabolism, stress and redox, lipid and secondary metabolism, signaling, and transport were also present in relatively high abundance [15,16,17].

### 2.3. Proteins Involved in Isoprenoid Biosynthesis and Accumulation

For the first time, LC-MS/MS was used as an analytical method for the identification of chromoplasts from saffron stigma at anthesis stage. We produced a proteomic draft, which represents a resource for future studies in saffron and organelle field. Proteomic tools have been recently applied to characterize the proteome of fresh and dried saffron stigmas and style in order to identify saffron from different geographical origins and to detect possible cases of fraud. In this study, 1D-Gel Electrophoresis was carried out and only five proteins have been identified. No differences were found among saffron from different location and the protein profile was quite distinct from the two common adulterants, *Carthamus tinctorius* and *Gardenia jasminoides* [18]. Since *Crocus sativus* has not been sequenced yet, data obtained from proteomes analysis were searched against the Magnoliophyta (2057887 sequences) and *Crocus* (513 sequences) databases from UniProtKB (available on: http://www.uniprot.org/). Analysis of saffron chromoplasts showed the presence of important enzymes involved in isoprenoid pathways as found the presence of the 1-deoxy-d-xylulose 5-phosphate synthase (DXS, Fraction 2) and isoreductoisomerase (DXR, Fractions 1 and 2) enzymes (Table S1). The first committed step in the carotenoid pathway is catalyzed by PSY, resulting in the condensation of two C-20 geranylgeranyl diphosphate (GGPP) molecules to form phytoene [19]. Only *Phytoene synthase 2* (*CsPSY2*) was detected in the proteome of the present study. Phytoene desaturase (PDS) and ζ-carotene desaturase (ZDS) were detected in F1 and F2 (Table 1 and Table 2) with PDS having the highest PSMs within all the enzymes of the pathway. 15-*cis*-ζ-carotene isomerase (Z-ISO) and the carotenoid isomerase (CRTISO) were identified in both fractions (Table 1 and Table 2). Both lycopene β-cyclase CstLcyB1 and CstLcyB2a proteins previously described [6] were present in the two fractions (Table 1, Table 2 and Table S1).

β-carotene hydroxylase CsBCH1 and CsBCH2 described for saffron were not found in F1 and only CsBCH1 was detected in fraction F2 (Table 2). We could not find any of the cytochrome P450 enzymes CYP97A3 or CYP97C1 member in the saffron proteome but, interestingly, a CYP97B2 enzyme, previously reported in *Arabidopsis* to be involved in xanthophyll synthesis [20], was found. All the Carotenoid cleavage dioxygenase enzymes (CCDs) were detected in the analyzed fractions with the exception of CCD8, expressed at low levels in saffron stigma [21], and CCD1 having a cytoplasmic location [14]. CCD2, the key enzyme in crocetin formation [9], is a plastidic enzyme [8] and was found in both fractions (Table 1). Similarly, also CCD4b, another plastid-localized enzyme, playing a key role in pollinator attraction [14], was present in both fractions (Table 1). The last carotenoid dioxygenase identified in the saffron proteome was CCD7. This enzyme, together with CCD8, displays a plastid distinct localization and is involved in strigolactone biosynthesis [22].

Other enzymes detected in the saffron proteome and involved in the apocarotenoid pathway were aldehyde dehydrogenases (ADHs) and UDP-dependent glycosyltransferases (UGTs). In addition to carotenoid metabolism, we found several proteins associated to the synthesis of other isoprenoids: tocochromanols and quinones (all present in Fraction 2).

### 2.4. Proteins of Interest in Sequestration and Related to Chromoplast Replication and Remodeling

Fibrillin and fibrillin homologs, known to be the main components of carotenoid-lipoprotein substructures, were identified (Table 2). Another fundamental player in carotenoid accumulation is the *Or* gene [23], which is not directly involved in carotenoid biosynthesis at transcript level, but can regulate PSY abundance at post-transcriptional level [24]. Within saffron proteome, several Or homologs were identified in F2.

Replication of chromoplasts is occasionally observed, such as in *Forsythia suspensa* petals [25] and pepper fruits [26]. Several homologs of the Plastid-dividing ring protein 3 and of the FtsZ proteins (Table S1) involved in this mechanism were detected. In addition, we found two members of the chloroplast inner envelope proteins (CIEPs) [27], as well the protein Chloroplast J-like domain 1, which affects plastid lipid composition and was shown to interact with CIEPs in the frame of the replication process [28].

### 2.5. Gene Expression Analysis of Genes Involved in Key Processes of Crocins Metabolism

The expression data of carotenogenic and sequestration genes detected in the chromoplast proteome, throughout the stigma development, are listed in Table 3 and Figure S1. The two master genes of the MEP pathway, *DXS1* and *DXR*, exhibited their highest peak of expression at anthesis stage. All the carotenogenic genes were expressed in all the developmental time-points except for *geranylgeranyl phyrophosphate synthase* (*CsGGPS*), *CsCCD7*, *CsCCD4b*, and *CstLcyB1*, whose transcripts were absent in one or more stages.

ADH activity is needed to catalyze the oxidation of the crocetin dialdehyde to crocetin; for this reason, we looked for ADHs in saffron chromoplasts, and the corresponding genes were identified in the transcriptome, thus allowing the comparison of these sequences with that previously characterized and recognizing apocarotenoids as substrates [29,30,31]. Overall, 12 different peptides were detected in the proteome analyses, and further utilized for the identification of the corresponding encoding gene sequences in the transcriptomes. Five different ADHs, ADHComp11367 (accession number KU577907) (Q9LLR2, W8SQV6 and M8AU02), ADHComp3898 (accession number KU577905) (A0A061E1Z5, A0A0B2PSM8, Q8RVW2 and W9RMTS), ADHComp54788 (accession number KU577904) (A0A059BY89 and I1QYB5), ADHComp20158 (accession number KU577906) (K9N4H5) and ADHComp2946 (accession number KU577903) (A0A072TS23 and Q9LRI6), were identified. Phylogenetic analyses revealed that one ADH (*ADHComp54788*) was closely related to *carD*, involved in the conversion of β-apo-4′-carotenal in β-apo-4′-carotenol [30], and showed its highest expression levels at anthesis, while the four others ADHs were more closely related to retinal aldehyde dehydrogenases [31,32], being *ADHComp11367* and *ADHComp2946* with the highest expression level at anthesis stage, whereas *ADHComp3898* and *ADHComp20158* at orange and white stages, respectively (Figure S2).

The action of glucosyltransferases is responsible for the conversion of crocetin into crocins. We investigated the expression of the two UGTs identified in our proteome analyses. Transcripts for UDP-glucosyltransferase *UGT85U1* and *CsUGT2* were detected from white stage, with the highest peak of expression at anthesis and red stages, respectively (Table 3).

Three different fibrillin-like proteins were detected after manual curation of the sequences; *PF2* and *PLAP13* showed the same pattern of expression, with a high level at the orange stage, while *PLAP2* was mostly up-regulated at the red stage (Table 3).

### 2.6. Gene Expression Analyses of Starch Metabolism Genes: The Carbohydrate Supply for Apocarotenoids Biosynthesis

In addition to carotenoid/transport genes, and due to the evidence by electron microscopy of relevant amount of starch in the cell plastids, we also checked the levels of some genes related to starch metabolism. The expression of glucose-pyrophosphorylase (UGP3), starch branching enzymes (SBE2.1/2.2), granule bound starch synthase (GBSS1) and starch synthase (SS2/3) were up-regulated during the earlier stages, reaching a maximum of expression during the yellow and orange stages, where the largest amounts of amyloplasts were detected, and then started to decline at the red stage. In contrast, the expression of amylase enzymes was down-regulated during the earlier stages and reached the highest expression during the anthesis stage, where no amyloplasts were observed (Table 3). All these results agreed with the gradual degradation of starch deposits observed during the stigma development.

### 2.7. Correlation Analyses to Identify Candidate Genes Involved in Apocarotenoid Production

**Figure 6:**
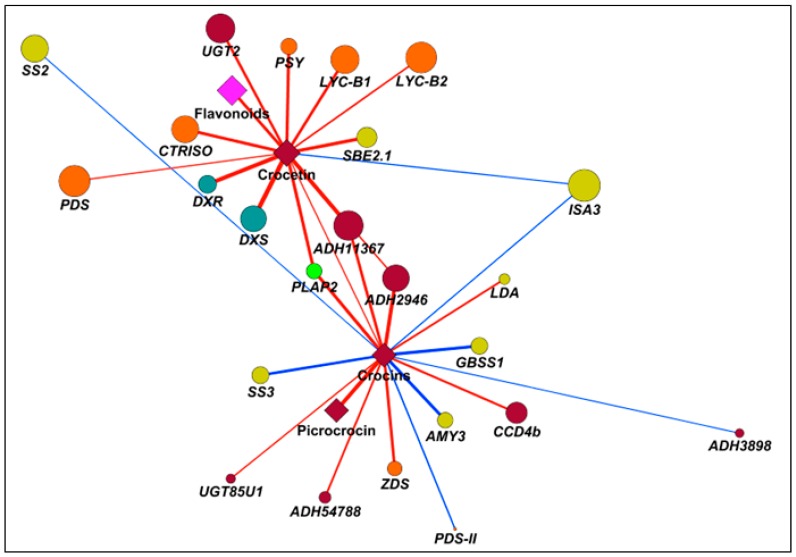
Correlation network of chromoplast genes/metabolites, involved in saffron apocarotenoid synthesis and transport. Genes and metabolites are represented as rounds and diamonds. Different colors identify genes/metabolites involved in MEP (turquoise), carotenoid (orange), apocarotenoid (burgundy), plastid development (green), and starch (yellow) biosynthesis. Blue and red edges refer, respectively, to negative and positive correlations; only correlations >|0.65| are shown. Edge width and node size were drawn according, respectively, the Pearson correlation coefficient (|*p*|) and the node strength (ns). Edge length was inversely proportional to |*p*|.

**Figure 7:**
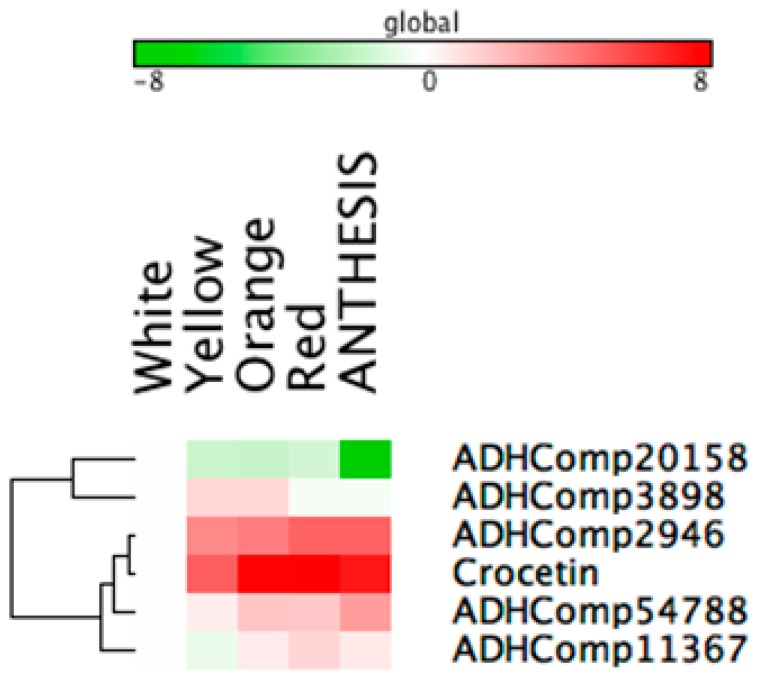
Hierarchical clustering (HCL) of crocetin and aldehyde dehydrogenase (ADH) found in the saffron proteome. Green and red squares represent the values of log2-transformed relative data with respect to the white stage according to the color scale shown. Hierarchical clustering was calculated on rows, applying the Pearson correlation coefficient with the average linkage algorithm.

To unravel regulative mechanisms underlying apocarotenoid accumulation in saffron stigma, we exploited correlation analyses on a set of variables including crocetin, crocins and picrocrocin, as well transcripts of carotenoid/apocarotenoid biosynthesis, plastid development and starch metabolism. Symmetric correlation matrix allowed investigation in the relationships within elements of different or of the same pathway (Figure S3). Overall, MEP/carotenoid transcripts, together with *CCD2* and *UGT2* but except for *PDS-II* and *ZDS*, exhibited a very high extent of positive correlations. Notably, the same genes were also correlated with the same sign towards a series of transcripts associated to plastid transformation (*PF2*, *PLAP2*, and *PLAP13*) and starch synthesis (*UGP3*, *SBE2.1*, and *SBE2.2*). Conversely, a second group of genes in the starch metabolism under study (synthetic: *SS2* and *SS3;* catabolic: *BAM3*, *ISA3*, *AMY3*, and *LDA*) displayed an area of general negative correlation with respect to most of the other transcripts analyzed. In the present study, we used correlation networks to highlight transcripts positively or negatively correlated with crocetin and crocins (Figure 6, Figures S4 and S5, Table S2). Crocetin only displayed positive correlations, except for *ISA3*, particularly yielded towards MEP (*DXS* and *DXR*) and carotenoid genes (PSY, *CRTISO*, and *LYC-B1/B2*), indicating a tight up-stream regulation exerted by isoprenoid metabolism on apocarotenoid synthesis. Interestingly, *UGT2*, known to utilize crocetin as substrate to produce crocins, also showed a significant positive correlation (0.81), and could be considered a positive control. Conversely, crocins did not share relevant correlations with early MEP/carotenoid genes, except for *ZDS* (positive, 0.81) and *PDS-II* (negative, −0.70), but were basically characterized by a series of Pearson coefficients >|0.65|: positive towards a carotenoid dioxygenase (*CCD4b*, 0.77), one aldehyde dehydrogenases (*ADH54788*, 0.75) and UGT85U1 (0.70); and negative in relation to a series of starch-related genes, ranging from *SS2* (−0.66) to *AMY3* (−0.84). These data suggested that crocins synthesis in saffron stigma is subjected to a regulative process, acting at chromoplast development and transformation levels, rather than at carotenoid biosynthetic time. We explored correlation analysis to reveal transcript/s coding for ADH activities, and responsible for the conversion of crocetin dialdehyde into crocetin. In this context, correlation networks highlighted two potential ADHs highly correlated with crocetin: *ADH2946* (0.71) and *ADH11367* (0.94). With the same objective, we exploited the Pearson squared distance algorithm in a Hierarchical Clustering (HCL) analysis (Figure 7) which confirmed the high level of relationship between crocetin and *ADH2946*/*ADH11367*, as well evidenced the presence of a third candidate (*ADH54788*) with an expression pattern like crocetin.

## 3. Discussion

Variations, at the cytological level, occurring along with the massive synthesis and accumulation of apocarotenoids, and especially the differentiation and evolution of chromoplasts throughout the development of saffron stigmas, have hardly been studied. In the present work, we studied the process of chromoplast differentiation in saffron during stigma maturation, together with the analyses of gene expression and proteomic profiling, in order to better understand the processes taking place in the chromoplasts at different stages of synthesis and accumulation of crocetin and crocin metabolites. In addition, we attempted to provide new insights into the possible transport mechanisms implicated in the release of these apocarotenoids into the central vacuole.

Protein profiling of the chromoplast revealed that the major metabolic pathways were associated to carbohydrate, amino acid, lipid and carotenoid metabolism, as well to protein regulation and transport, and oxidative stress. A number of Calvin cycle proteins were detected in saffron chromoplasts. The persistence of a Calvin cycle and oxidative pentose phosphate pathway proteins (i.e., transketolase) is thought to provide intermediates for glycolysis and a main source of reducing power for various processes, respectively, in chromoplasts. These proteins have been detected in several chromoplasts analyzed [33]. In addition, various glycolysis enzymes were observed, suggesting an active metabolism in chromoplasts to generate energy and precursors for the synthesis of metabolites. Enzymes involved in fatty acid metabolism and amino acid as well as many components involved in maintaining cell redox homeostasis, were also found and confirm the chromoplast capacity in relation to lipid synthesis, which could be also responsible for the massive generation of vesicles, as well to chromoplast protection, by regulating redox status and reactive oxygen species (ROS).

The analysis of chromoplasts from carotenoid enriched tissues of saffron stigma identified several MEP and early carotenoid pathway enzyme proteins. Correlation analysis showed that crocetin synthesis is highly dependent on carotenoid pathway, while the process of crocin accumulation looked more related to the conversion of the amyloplasts in chromoplasts. Despite of the main function of chromoplasts which is the accumulation of pigments, a high number of compounds are synthetized in these structures such as sugars, starches, lipids, aromatic compounds, vitamins and hormones [34,35]. Chromoplasts, unlike green plastids, are unable to synthesize all the metabolites necessary for these processes and, thus, need often to import them by the cytosol. To sustain the biosynthetic activities, sugars and energy (ATP) are required [13]. Sugars can be imported from the cytosol [36], or can be taken from starch degradation [13,37]; [34]. Our results indicate that in the earlier stages of style development from white to yellow-orange an accumulation of membranous sinks and starch granules occur, which are more abundant in the yellow stage, decreasing hereinafter. However, these structures are present in all stages excluding the anthesis stage.

Carotenoid accumulation in chromoplast has been attributed to a variety of factors, among them, important roles are played by specific proteins called chromoplast-specific carotenoid-associated proteins [38] and the Orange (Or) protein [39]. Eight proteins belonging to the fibrillin/plastid lipid associated protein family were detected in saffron chromoplasts and were developmentally regulated with carotenoid content. These proteins are the main component of carotenoid lipoprotein sequestration and are implicated in the over-production of pigments due to a sink effect. Transgenic tomato plants expressing pepper fibrillin were found to accumulate this protein during fruit ripening from an early mature green stage, and having a two-fold increase in carotenoids content and derivatives [40]. We found a very strong correlation between *PLAP2* and crocetin and crocins, suggesting the involvement of this protein in the assembly of these apocarotenoids in macromolecular structures of the chromoplasts. In addition, these proteins have been reported to be involved in abiotic stress responses [41]; in this context, their accumulation in saffron chromoplast could be interpreted as a further mechanism of organelle protection.

Chromoplasts are organelles with a highly active metabolic system accumulating different kinds of carotenoid pigments in different types of structures but the relationship between the structures and the nature of the chemical compounds contained inside these vesicles is not always clear. In many chromoplasts, a special membrane system, called system of internal membranes of chromoplasts (MICs), is formed de novo by invagination of the inner membrane of the plastid where carotenogenic enzymes are found and biosynthesis of carotenoids takes place [42,43]. This hypothesis is supported by the presence, in the saffron proteome, of proteins as CIEPs Chloroplast J-like domain 1, which can contribute to the generation of MICs, vesicles and to plastid division. Taken together our results suggest that crocetin synthesis happen in the saffron chromoplasts MICs, and mainly from the orange stage onwards. Glycosylation of crocetin should happen also in the chromoplast, due to the plastidial localization of CsUGT2.

**Figure 8:**
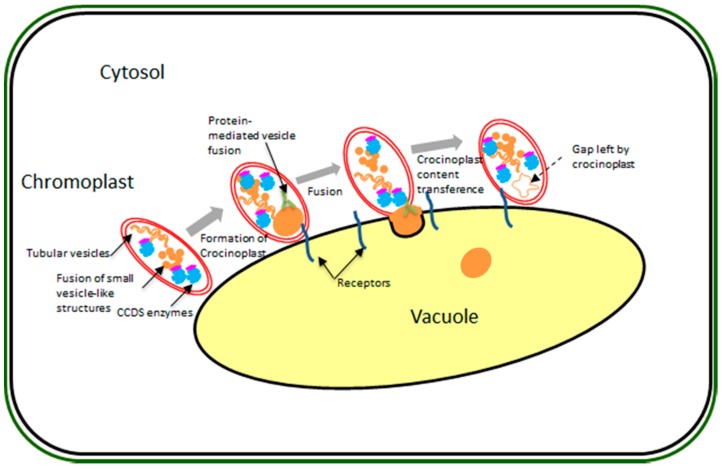
Proposed model of crocin transport from chromoplast to the vacuole.

From the generally polarized MICs, the formation of large globules resembling lipid droplets, and much larger than plastoglobules, occur from the red stage onwards. Chromoplasts containing these large globules (hereafter named crocinoplast) are usually close or connected to the vacuole, suggesting that these lipids droplet-like structures could contain saffron crocins. Our model consists of a direct transference from the polarized end of the chromoplast, where the formation of crocinoplasts containing crocins occurs, (Figure 2 and Figure 8) to the vacuole. They might originate from many smaller vesicle-like structures that gradually fuse together. Direct apocarotenoid transport from chromoplast to vacuole was observed in *Bixa orellana* [44] and fusion between carotenoid rich lipid drops and the vacuole has been described in aging spores of *Puccinia distincta* [45]. Our observations suggested that, in some cases, the whole chromoplast is digested by the vacuole. Processes of macroautophagy, whereby plastids were introduced into lytic vacuoles to be digested have been previously described in *Brassica napus* embryogenic microspores [46].

Understanding the biosynthesis, transport and storage processes of crocins fills the gap in the current knowledge regarding the metabolism of these apocarotenoids and benefits metabolic engineering which could use these compounds in heterologous crops for improving the nutritional quality of plant-based foods.

## 4. Materials and Methods

### 4.1. Light and Transmission Electron Microscopy

Stigmas were obtained from *Crocus sativus* grown under field conditions in the Botanical Garden of CLM (Albacete, Spain). We collected samples of saffron stigmas covering all the developmental stages, including the white, yellow, orange-yellow, orange, red and red-scarlet [14]. Basically, fresh stigmas from saffron plants were excised, immediately transferred to aluminum sample holders, cryoprotected with 150 mM sucrose, frozen in a Leica EM HPM-100 high-pressure freezer (Leica Microsystems, Vienna, Austria), and then transferred to LN_2_. Subsequently, samples were processed as previously described by [47]. In brief, samples were freeze substituted with anhydrous acetone + 2% OsO_4_ at −80 °C for 5 days, followed by slow warming to room temperature over a period of 24 h. After rinsing in several acetone washes, they were removed from the holders and infiltrated with increasing concentrations of Spurr resin (Ted Pella, Redding, CA, USA) in acetone according to the following schedule: 4 h in 2% resin, 4 h in 5% resin, 12 h in 10%, 25%, 50%, 75% resin and 36 h in 100% resin. We obtained poor quality freezing stigmas tissues using high pressure freezing, for this reason, they were chemically fixed in Karnovsky´s fixative (4% formaldehyde + 5% glutaraldehyde in 0.025 M cacodylate buffer, pH 6.9) for 5 h, postfixed in 1% OsO_4_ in cacodylate buffer, dehydrated in ethanol series and embedded in Spurr resin using a Leica EM TP automated tissue processor.

For both high pressure, frozen and chemically fixes samples, resin polymerization was performed at 70 °C for 1 day. Once plastic blocks were produced, thin sections (1 μm) were obtained for light microscopy observation, and ultrathin (~80 nm) sections were obtained for electron microscopy, using a Leica UC6 ultramicrotome in all the cases. Thin sections were observed unstained under phase contrast in a Nikon Eclipse E1000 microscope (Nikon, Tokyo, Japan). Ultrathin sections were mounted on formvar-coated, 200 mesh copper grids, stained with uranyl acetate and lead citrate, and observed in a Jeol JEM 1010 transmission electron microscope (Jeol, Tokyo, Japan).

### 4.2. Isolation and Purification of Saffron Chromoplasts from Fresh Stigmas

For chromoplast isolation, three biological replicates were processed and analyzed independently. Chromoplasts were isolated from fresh saffron stigmas at the red stage (about 30 g) following the method described by Angaman et al. [48]. Briefly, 30 g stigmas were mixed with 60 mL of buffer A (100 mM Tris-HCl pH 8.2, 0.33 M sorbitol, 2 mM MgCl_2_, 10 mM KCl, 8 mM EDTA, 10 mM ascorbic acid, 5 mM l-cysteine, 1 mM PMSF, 1% PVPP and 1 mM DTT). Then after, the obtained purée was homogenized with a Waring blender and filtered through 8 layers of gauze and then through 2 layers of cheesecloth. The homogenate was mixed and centrifuged for 2 min at 200× *g*. The supernatant was recovered and centrifuged for 10 min at 5000× *g*. The obtained pellet was resuspended in 50 mL of buffer B (buffer A without PVPP) and centrifuged for 10 min at 5000× *g*. The pellet was resuspended in 4 mL of buffer B and chromoplasts were fractionated by ultracentrifugation on a discontinuous sucrose gradient (15%, 30%, 40%, 50% and 60% in Tris-HCl pH 7.4 supplemented with 1 mM DTT) for 1 h at 100,000× *g*. Chromoplast fractions were recovered by gentle aspiration with a Pasteur pipette. The collected fractions were washed with one volume of buffer B and chromoplasts were recovered by centrifugation (10 min, 5000× *g*).

### 4.3. Extraction and Analysis of Crocins by HPLC-DAD, Marker-Enzyme Assays and Sequence Analysis

The collected fractions were extracted with 1 mL Tris-HCl (50 mM, pH 7.5; containing 1 M NaCl), and incubated for 10 min on ice. One volume of CHCl_3_ was then added, mixed, and the extract incubated on ice for an additional 10 min followed by centrifugation at 3000× *g* for 5 min at 4 °C. The lower CHCl_3_ phase was evaporated under N_2_ gas and the dried residues were stored together with the upper aqueous phases at −80 °C until analysis by HPLC. All assays were performed in triplicate. The HPLC methods used for the analysis and detection of glycosylated apocarotenoids and carotenoids have been previously described [4,14].

The activity of the enzymes succinate dehydrogenase, catalase and alcohol dehydrogenase was measured in purified chromoplast samples (resuspended in buffer B). Succinate dehydrogenase and catalase activities were measured in samples previously treated with 1% Triton X-100 for 10 min at 4 °C. The assay methods used for enzyme activities were those described by [48]. Marker-enzyme assay determinations were done in duplicate using two independent chromoplast preparations. MapMan Bins (available on: http://mapman.mpimp-golm.mpg.de) and Venn diagram (available on: http://bioinformatics.psb.ugent.be/webtools/Venn/) were used for functional assignments and for comparison, respectively.

### 4.4. LC-MS/MS as an Analytical Method for the Identification of Chromoplast Proteins

Protein samples were processed and analyzed by nanoLC-MS/MS. In brief, salts and non-protein components were removed prior to proteomic analysis by means of a short electrophoretic run (10 min). The protein band was excised, cut into small pieces and destained with 50 mM ammonium bicarbonate/50% ACN (acetonitrile), dehydrated with ACN and dried. Samples were reduced by adding dithiothreitol to a final concentration of 10 mM and alkylated with iodoacetamide to a final concentration of 50 mM. Then, gel pieces were dried, rehydrated with 12.5 ng/µL trypsin in 50 mM ammonium bicarbonate and incubated overnight at 30 °C. Peptides were extracted at 37 °C using ACN and 0.5% TFA, and then dried, cleaned using ZipTip with 0.6 µL C18 resin (Millipore, Darmstadt, Germany) and reconstituted in 5 µL 0.1% formic acid/2% ACN prior to MS analysis. All peptide separations were carried out on a NanoEasy HPLC (Proxeon Biosystems, Odense, Denmark) coupled to a nanoelectrospray ion source (Proxeon Biosystems). Six microliters of each sample were loaded onto a C18-A1 ASY-Column 2 cm precolumn (Thermo Scientific, Madrid, Spain) and then eluted onto a Biosphere C18 column (C18, inner diameter 75 µm, 15 cm long, 3 µm particle size, Nano Separations, Nieuwkoop, The Netherlands). The mobile phase flow rate was 250 nL/min, and 0.1% formic acid, 2% ACN in water (solvent A) and 0.1% formic acid and 100% ACN (solvent B) were used as eluents. The gradient profile was set as follows: 0%–45% solvent B for 130 min, 45%–95% solvent B for 15 min and 5 min isocratically at 95%. Mass spectra were acquired on the LTQ-Orbitrap Velos in the positive ion mode. Full-scan MS spectra (*m*/*z* 300–1900) were acquired in the Orbitrap with a target value of 1,000,000 at a resolution of 60,000 and the 15 most intense ions were selected for collision induced dissociation (CID) fragmentation in the LTQ with a target value of 10,000 and normalized collision energy of 35%.

Mass spectra * raw files were searched against the Magnoliophyta (2,057,887 sequences) and Crocus (513 sequences) databases from UniProtKB (available on: http://www.uniprot.org/) using the SEQUEST search engine through Proteome Discoverer (version 1.3.0.339, Thermofisher, Madrid, Spain) and then the results were merged and checked manually for further analyses to avoid redundancy. Precursor and fragments mass tolerance were set to 10 ppm and 0.8 Da, respectively. Search parameters included a maximum of two missed cleavages allowed, carbamidomethylation of cysteines as a fixed modification and oxidation of methionine as a variable modification. Peptides were validated through the algorithm Percolator (FDR 0.01) [49] and only those with high and medium confidence were admitted. Protein identifications were accepted if they contained at least two identified peptides, except for proteins involved in the crocins pathway. CD-HIT web server (available on: http://weizhong-lab.ucsd.edu/cdhit_suite/cgi-bin/index.cgi?cmd=cd-hit) was used to search for sequences redundancy with a sequence identity cut-off of 0.9.

### 4.5. Gene Expression by Quantitative Reverse Transcription-PCR (qRT-PCR)

The gene-specific oligonucleotides used for expression analysis are listed in Table S3. The qRT-PCR was carried out on cDNA from five developmental stages selected from stigma at the white, yellow, orange, red and anthesis stages. Three biological replicates were achieved and reactions were set up in a final volume of 25 μL in GoTaq^®^ qPCR Master Mix (Promega, Madison, WI, USA) according to manufacturer’s instructions. The constitutively expressed 18SrRNA gene was used as a reference gene. The cycling parameters were used as described in [21].

### 4.6. Bioinformatic Analysis

Heat map, hierarchical clustering (HCL) and correlation matrix were drawn by using GENE-E (available on: http://www.broadinstitute.org/cancer/software/GENE-E/), while correlation networks were performed as previously described [50] with slight modifications: force-directed layout was used, as well as visualization using crocetin and crocins as central hubs and building edge length inversely proportional to the Pearson correlation coefficient (|*p*|).

## 5. Conclusions

Crocins, the glucosides of crocetin, are present at high concentrations in the stigmas of saffron. Crocins biogenesis starts with zeaxanthin cleavage by CCD2, followed by the activity of an uncharacterized ADH, and is further glucosylated by CsUGT2. The generated crocins accumulate in the vacuole. Variations at the cytological level that go along with the massive synthesis and accumulation of these apocarotenoids, and especially the differentiation and evolution of chromoplasts throughout the development of saffron stigmas, have hardly been studied. The data obtained by monitoring chromoplast development during stigma growth by means of transmission electron microscopy revealed the presence of a membranous network spanning the entire plastid volume together with the presence of plastoglobules defined the conversion of plastids into chromoplasts. Our data showed a direct transfer from large globule (crocinoplast) to the central vacuole in the later developmental stages. A first draft of the stigma proteome was depicted, which allowed the identification of several well-known plastid proteins and new candidates involved in crocetin metabolism. Results from correlation analyses on a set of variables including three apocarotenoid metabolites (crocetin, crocins and picrocrocin), as well transcripts of carotenoid/apocarotenoid biosynthesis, plastid development and starch metabolism, showed a coordinate regulation in the expression of pathways boosting carotenoid and apocarotenoid synthesis and involved in the chromoplast formation and development. The data obtained from this study represent a resource for a better comprehension of saffron physiology and metabolism.

## Figures and Tables

**Table 1:** Identification of proteins involved in carotenoid pathway and plastid-lipid-associated proteins, deduced from LC-MS/MS data of fraction F1 from saffron chromoplasts.

Uniprot ID	Homologous to Predicted Proteins	Score	Cov. (%)	# Prot.	# UP.	# Pept.	# PSMs.
**Carotenoid Pathway**							
H9MZJ1	1-Deoxy-d-xylylose 5-phosphate reductoisomerase	2.9	3.9	20	1	1	1
J9XGW7	4-Hydroxy-3-methylbut-2-enyl diphosphate reductase	5.56	6.74	71	1	2	2
W8SQV6	Aldehyde dehydrogenase 2-1	74.4	8.6	109	1	4	15
A0A061E1Z5	Aldehyde dehydrogenase 6B2	40.4	10.0	34	3	4	10
M8AU02	Aldehyde dehydrogenase family 2 member B4	28.0	5.7	34	1	4	8
A0A0B2PSM8	Aldehyde dehydrogenase family 2 member B7	50.3	11.0	156	2	6	16
Q9LLR2	Aldehyde dehydrogenase	**111.3**	10.1	99	1	3	**25**
A0A075W695	Carotenoid cleavage dioxygenase 2	29.0	17.3	34	2	8	9
A0A075IEG5	Carotenoid cleavage dioxygenase 7	2.6	2.7	1	1	1	1
Q2R2A6	Carotenoid isomerase	48.2	5.6	27	2	3	11
B7SNW3	Chromoplast carotenoid cleavage dioxygenase 4b	11.3	7.9	5	4	4	4
D2IFC2	Chromoplast-specific lycopene β cyclase OS=Crocus sativus	80.4	13.4	1	4	4	18
Q6X1C0	Crocetin glucosyltransferase 2	24.8	10.6	18	4	4	6
A0A0D4D933	CRTISO	60.7	7.7	70	1	4	14
S5VPK3	Lycopene β-cyclase	8.1	10.8	65	1	1	2
B9S6H4	Phytoene dehydrogenase	16.7	2.0	1	1	1	4
Q84XU1	Phytoene desaturase	**105.4**	25.5	229	10	10	**26**
P37273	Phytoene synthase 2	9.9	3.5	16	1	1	3
C5I849	Phytoene synthase	17.0	5.0	1	1	1	3
A0A075M6P3	UDP-glucosyltransferase UGT85U1	3.7	4.0	2	1	1	1
I0AXV3	ζ-Carotene desaturase	62.5	15.5	113	2	7	15
D7TUM8	ζ-Carotene desaturase	55.7	11.6	88	1	5	14
**Plastid-Lipid-Associated Proteins**							
K7UIX3	Plastid-lipid-associated protein 2	15.1	9.5	19	1	2	4
A0A0B2RM96	Plastid-lipid-associated protein	39.0	7.8	24	1	2	9
A9CSJ8	Putative plastid lipid-associated protein	**42.7**	12.2	28	0	2	**10**

Proteins were searched against the Magnoliophyta and Crocus databases of Uniprot. Proteins are ordered alphabetically within each functional group, and the hits with maximal scores and number of PSMs in each category are highlighted in bold. The hits identified as homologous to *Crocus sativus* proteins are underlined. Cov. = coverage; # Prot. = number of proteins; # UP. = number of unique peptides; # Pept. = number of peptides; # PSMs. = number of peptide spectrum matches.

**Table 2:** Identification of proteins involved in carotenoid pathway and plastid-lipid-associated proteins, deduced from LC-MS/MS data of fraction F2 from saffron chromoplasts.

Uniprot ID	Homologous to Predicted Proteins	Score	Cov. (%)	# Prot.	# UP.	# Pept.	# PSMs.
**Carotenoid Pathway**							
A0A059VDA9	1-Deoxy-d-xylulose 5-phosphate reductoisomerase	39.0	24.3	95	1	7	10
G8FL07	1-Deoxy-d-xylulose 5-phosphate reductoisomerase	30.0	13.6	18	1	5	8
A0A075EAM0	1-Deoxy-d-xylulose 5-phosphate reductoisomerase	35.6	16.0	18	1	4	9
B9RB24	1-Deoxy-d-xylulose 5-phosphate reductoisomerase, chloroplast	15.1	4.8	3	1	2	4
W6HY22	Chloroplast 1-deoxy-d-xylulose-5-phosphate reductoisomerase	49.3	24.4	133	1	7	13
C7U110	1-Deoxy-d-xylulose-5-phosphate synthase 1	10.8	4.6	99	1	3	4
O22567	1-Deoxy-d-xylulose-5-phosphate synthase 1, chloroplastic	11.1	4.6	94	1	3	4
O81014	4-Diphosphocytidyl-2-C-methyl-d-erythritol kinase, chloroplastic	3.9	6.75	10	1	1	1
P93841	4-Diphosphocytidyl-2-C-methyl-d-erythritol kinase, chloroplastic/chromoplastic	2.8	4.81	6	1	1	1
J9XH53	Putative 4-diphosphocytidyl-2-C-methyl-d-erythritol kinase	3.8	5.24	1	1	1	1
D2D5E3	(*E*)-4-hydroxy-3-methylbut-2-enyl diphosphate synthase	55.3	13.78	53	1	7	15
W8SNM0	4-Hydroxy-3-methylbut-2-en-1-yl diphosphate synthase protein	54.2	10.95	72	1	7	15
A0A089G093	1-Hydroxy-2-methyl-2-(*E*)-butenyl-4-diphosphate reductase	46.3	13.61	76	2	6	12
W5GTY8	2-C-Methyl-d-erythritol 2,4-Cyclodiphosphate synthase	8.2	18.01	80	3	3	3
D2CFT5	Hydroxymethylbutenyl diphosphate reductase	22.1	12.31	92	2	6	6
Q9SLG2	GGPP4_ARATH Geranylgeranyl pyrophosphate synthase 4	3.4	4.64	1	1	1	1
A0A097PPW4	15-*cis*-ζ-carotene isomerase	7.1	4.5	1	1	1	2
A0A061E1Z5	Aldehyde dehydrogenase 6B2	40.4	11.3	36	2	4	8
Q9LRI6	Aldehyde dehydrogenase ALDH2a	32.9	6.0	22	1	3	9
M8AU02	Aldehyde dehydrogenase family 2 member B4	51.2	5.7	27	1	4	14
W9RMT5	Aldehyde dehydrogenase family 2 member	34.1	6.4	91	1	3	9
A0A059BY89	Aldehyde dehydrogenase	13.2	7.7	85	3	3	4
I1QYB5	Aldehyde dehydrogenase	4.2	2.3	4	1	1	1
Q8VXP2	β-Carotene hydroxylase	2.5	4.9	1	1	1	1
A0A075W695	Carotenoid cleavage dioxygenase 2	72.6	23.7	1	4	12	18
B7SNW1	Carotenoid cleavage dioxygenase 2	58.6	20.8	1	2	10	15
V5K7G6	Carotenoid isomerase (Fragment)	**152.3**	7.7	44	1	3	**34**
A0A072VJ38	Carotenoid isomerase	20.7	3.7	20	1	2	6
B7SNW3	Chromoplast carotenoid cleavage dioxygenase 4b	25.9	14.2	6	6	6	7
D2IFC2	Chromoplast-specific lycopene β cyclase	**128.5**	22.0	1	7	7	**29**
Q6X1C0	Crocetin glucosyltransferase 2	17.8	10.6	18	4	4	5
M7ZM48	Cytochrome P450 97B2, chloroplastic	3.34	3.81	16	1	1	1
F1BPW8	Lycopene β-cyclase	2.9	1.8	6	1	1	1
S5VPK3	Lycopene β-cyclase	19.1	10.8	65	1	1	5
K9N4H5	Mitochondrial aldehyde dehydrogenase 2B8	68.2	7.8	155	1	5	20
A0A072TS23	NAD-dependent aldehyde dehydrogenase family protein	28.4	5.5	102	1	3	8
Q84XU1	Phytoene desaturase	**285.3**	48.0	242	17	17	**68**
I1U6I7	Phytoene synthase	3.6	6	4	1	1	1
A0A097PKY8	Phytoene synthase	12.8	6.7	195	1	2	4
C5I849	Phytoene synthase protein	33.8	8.3	138	1	2	7
Q52QW5	Phytoene synthase, chloroplastic	35.2	5.8	153	1	2	10
A0A075M6P3	UDP-glucosyltransferase	19.7	4.0	2	1	1	5
I7H187	ζ-Carotene desaturase	21.6	9.3	1	1	2	5
I0AXV3	ζ-Carotene desaturase	**139.2**	21.8	106	5	11	**34**
C3VEQ0	ζ-Carotene desaturase, chloroplastic/chromoplastic	63.0	12.6	23	2	6	16
**Plastid-Lipid-Associated Proteins**							
O99019	Chromoplast-specific carotenoid-associated protein	12.3	10.4	1	1	1	3
I1MFL4	Chromoplast-specific carotenoid-associated protein	**82.8**	7.8	24	2	2	**19**
R9TH24	Chromoplast-specific carotenoid-associated protein	19.2	11.2	1	2	2	4
Q8S9M1-6	Isoform 6 of Probable plastid-lipid-associated protein 13, chloroplastic	2.4	5.7	21	1	1	1
A6XBI6	Plastid fibrillin 2	7.1	4.4	7	1	1	2
K7UIX3	Plastid-lipid-associated protein 2	14.7	9.5	19	2	2	4
A0A075F1U0	Constitutive plastid-lipid associated protein	16.5	28.9	48	2	3	5
K7UIX3	Plastid-lipid-associated protein 2	14.7	9.5	19	2	2	4

Proteins were searched against the Magnoliophyta and Crocus databases of Uniprot. Proteins are ordered alphabetically within each functional group, and the hits with maximal scores and number of PSMs in each category are highlighted in bold. The hits identified as homologous to *Crocus sativus* proteins are underlined. Cov = coverage. # Prot = number of proteins. # UP = number of unique peptides. # Pept = number of peptides. # PSM s= number of peptide spectrum matches.

**Table 3:** Transcript levels of genes involved in carotenoid pathway, and Plastid-lipid-associated proteins, at five different stages of stigma development. For more details, see materials and methods.

Gene	Pathway	Stigma Developmental Stage				
		White	Yellow	Orange	Red	Anthesis
*DXS*	MEP	245.6	282.5	795.7	1422.9	353.43
*DXR*	MEP	58.6	56.5	119.6	282.1	26.97
*GGPS*	MEP	0	11.0	51.7	13.8	0
*PSY*	Carotenoid	35.9	112.5	103.5	193.0	44.39
*PDS*	Carotenoid	52.3	76.3	228.0	189.6	46.18
*PDS-II*	Carotenoid	4.3	8.97	10.5	0	4.44
*ZISO*	Carotenoid	110.23	135.0	319.5	186.9	217.99
*ZDS*	Carotenoid	103	139.0	436.3	568.9	1257.168
*CTRISO*	Carotenoid	56	378.1	560.4	771.5	145.57
*LYC-B1*	Carotenoid	0	0	16.3	17.3	0
*LYC-B2*	Carotenoid	63.5	111.9	465.8	407.2	55.4
*BCH*	Carotenoid	8.7	39.4	118.2	70.6	44.28
*CCD2*	Apocarotenoid	93.8	307.5	693.8	594.2	15.83
*UGT2*	Apocarotenoid	68.2	943.6	1567.2	1935.4	256.6
*UGT85U1*	Apocarotenoid	62.1	218.9	688.9	541.6	1579.1
*CCD7*	Apocarotenoid	0	0	23.5	0	0
*CCD4b*	Apocarotenoid	0	0	0	11.7	46.5
*ADHComp2946*	Apocarotenoid	17.6	254.6	321.4	575.2	561.6
*ADHComp20158*	Apocarotenoid	171.2	60.4	54.5	69.7	0
*ADHComp3898*	Apocarotenoid	34.5	90.3	90.9	29.1	29.6
*ADHComp54788*	Apocarotenoid	35.5	57.4	134.8	132.5	319.6
*ADHComp11367*	Apocarotenoid	37.3	25.4	61.6	101.4	66.2
*GLUCOSEPYROPHOSPHORYLASE (UGP3)*	Starch	80.1	207.4	196.9	166.7	29.3
*STARCH BRANCHING ENZYME (SBE2.1)*	Starch	8.4	16.3	23	40.7	3.2
*STARCH BRANCHING ENZYME (SBE2.2)*	Starch	14.2	26.5	38.8	24.8	0
*GRANULE BOUND STARCH SYNTHASE (GBSS1)*	Starch	56.4	116.2	108	25.9	4
*STARCH SYNTHASE (SS2)*	Starch	60.3	41.1	0	9.5	0
*STARCH SYNTHASE (SS3)*	Starch	58.9	58.5	32	31.4	8
*B-AMILASE3*	Starch	4.8	5	23.5	14.4	74.6
*ALFA AMILASE (ISA3)*	Starch	19.7	16	9.4	9.9	10
*ALFA AMILASE (AMY3)*	Starch	2.9	6	7.1	0	0
*ALFA AMILASE (LDA)*	Starch	4.2	9.9	0	12.3	14.6
*Plastid fibrillin 2*	Plastid development	20.5	62.1	146.3	95	75.3
*Plastid-lipid-associated protein 2*	Plastid development	4.1	17	0	117	50.6
*Isoform 6 of Probable plastid-lipid-associated protein 13*	Plastid development	13.6	41.2	161.4	94.6	6.8

## Supplementary Materials

Supplementary materials can be found at www.mdpi.com/1422-0067/18/1/76/s1.
