# Supplementary material for: Unraveling Massive Crocins Transport and Accumulation through Proteome and Microscopy Tools during the Development of Saffron Stigma
Source: Int J Mol Sci. 2017 Jan 1;18(1):76. doi: 10.3390/ijms18010076 (PMC5297711; doi:10.3390/ijms18010076)
Supplement: Supplementary file 1 [file ijms-18-00076-s001.zip › ijms-165830-Supplementary Materials/ijms-165830-supplementary-for publication.pdf]

Supplementary Material: Unraveling Massive Crocins Transport and Accumulation through Proteome and Microscopy Tools during the Development of Saffron Stigma

Lourdes Gómez-Gómez, Verónica Parra-Vega, Alba Rivas-Sendra, Jose Maria Seguí-Simarro, Rosa Victoria Molina, Claudia Pallotti, Ángela Rubio-Moraga, Gianfranco Diretto, Alicia Prieto and Oussama Ahrazem

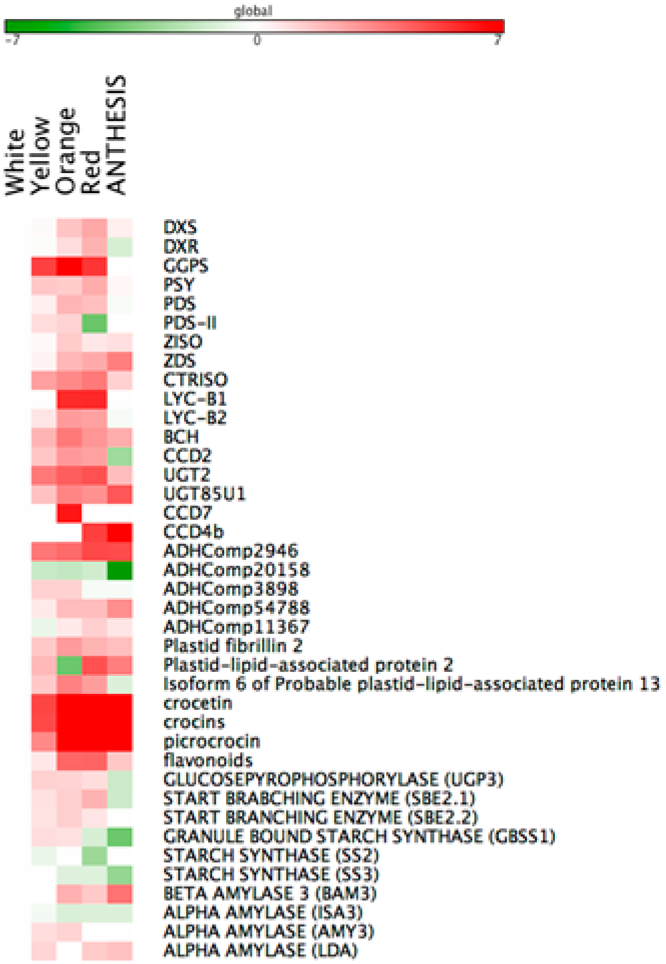

Figure S1. Heatmap of chromoplast genes/metabolites involved in saffron apocarotenoid synthesis. Green and red squares represent the values of log2-transformed relative data with respect to the white stage according to the color scale shown.

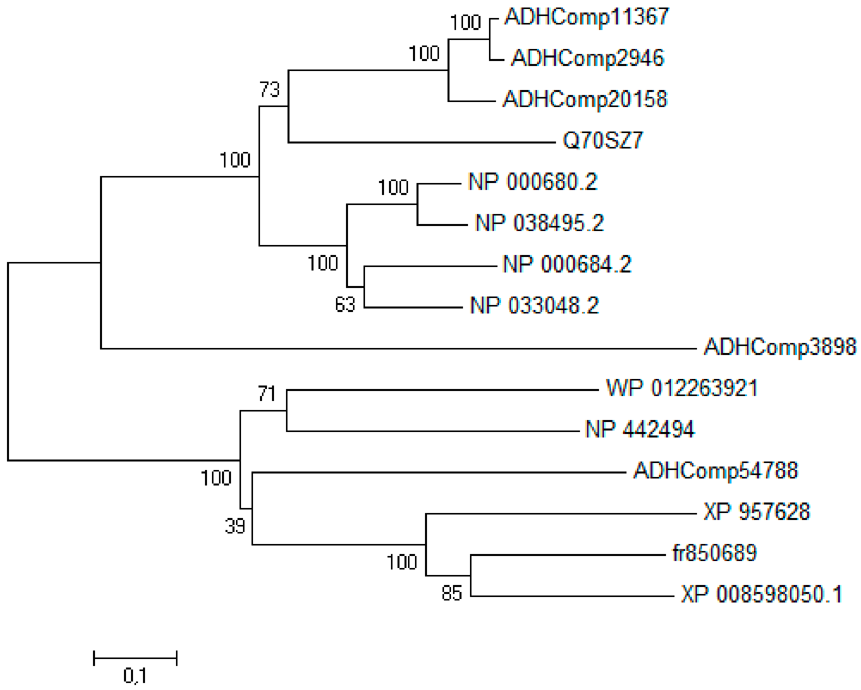

Figure S2. Dendrogram of several ADHs, obtained by using the Neighbor-Joining method. The sequences used were: huADH1A2, NP_000684.2 (*Homo sapiens*); huADH1A1, NP_000680 (*Homo sapiens*); huADH1A3, NP_033048.2 (*Homo sapiens*); ADH1, NP_038495.2 (*Mus musculus*); CsADH, Q70SZ7 (*Crocus sativus*); ADH, WP 010873792 (*Synechocystis sp.*); MaADH, WP 012263921 (*Microcystis aeruginosa*); CarD, fr850689 (*Gibberella fujikuroi*); BbADH3B1 (*Beauveria bassiana*); NcADH (*Neurospora crassa*); ADHComp2946, ADHComp20158, ADHComp3898, ADHComp54788 and ADHComp11367 (*Crocus sativus*).

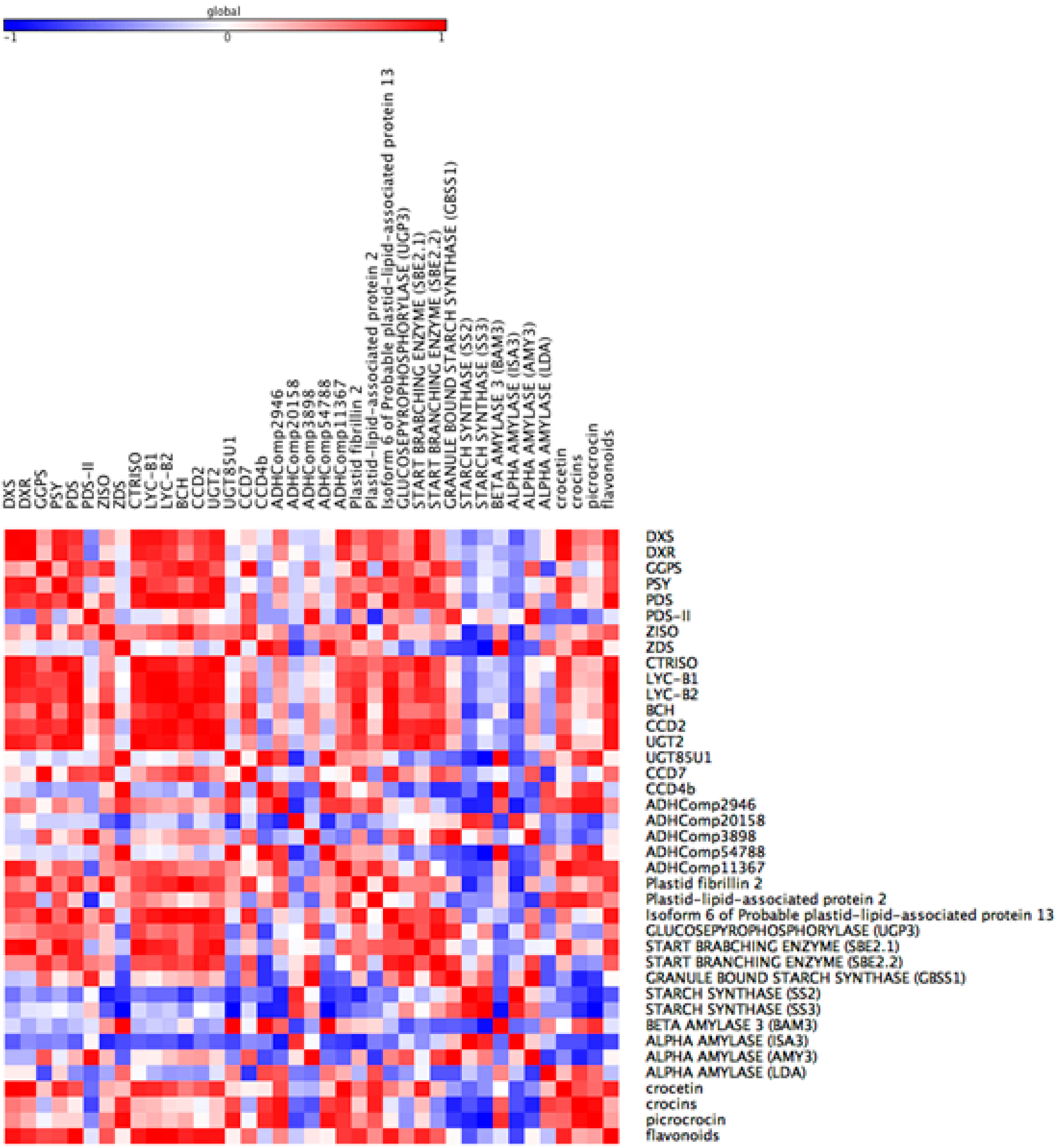

Figure S3. Correlation matrix of chromoplast-related transcripts and metabolites in the saffron stigma. Squares represent the correlation between the gene/metabolite heading the column and the gene/metabolite heading the row. Correlation analysis was carried out by calculating the Pearson correlation coefficient for each data pair and visualized using the GENE-E software. Pearson (|*p*|) values are shown and highlighted with different shades of red (positive correlations) or blue (negative correlations).

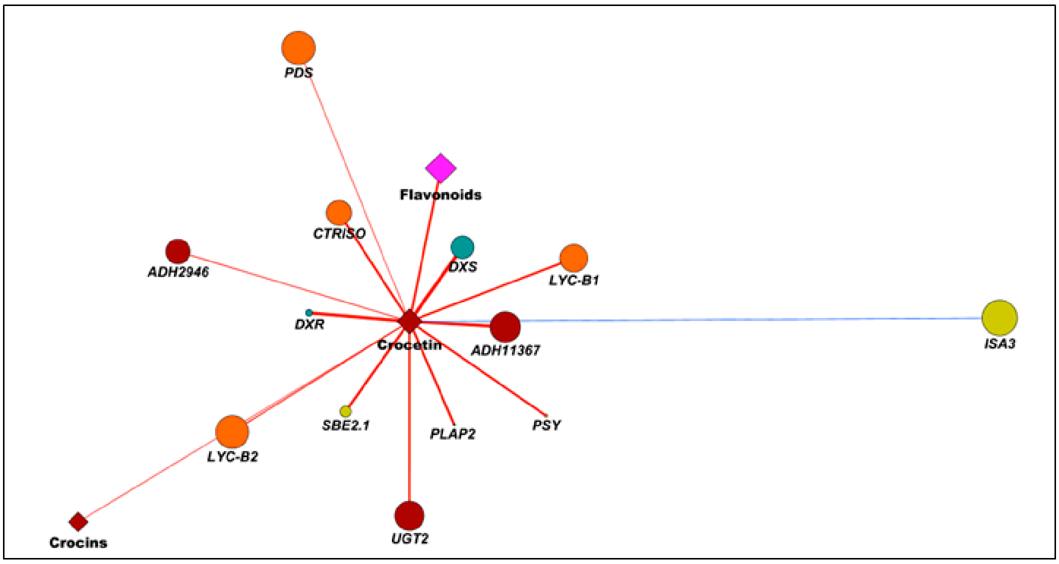

Figure S4. Correlation network of saffron chromoplast-related genes/metabolites using crocetin as central hub. Genes and metabolites are represented as rounds and diamonds. Different colors identify genes/metabolites involved in MEP (turquoise), carotenoid (orange), apocarotenoid (burgundy), plastid development (green), and starch (yellow) biosynthesis. Blue and red edges refer, respectively, to negative and positive correlations; only correlations >|0.65| are shown. Edge width and node size were drawn according, respectively, the Pearson correlation coefficient (|p|) and the node strength (ns). Edge length was inversely proportional to |p|.

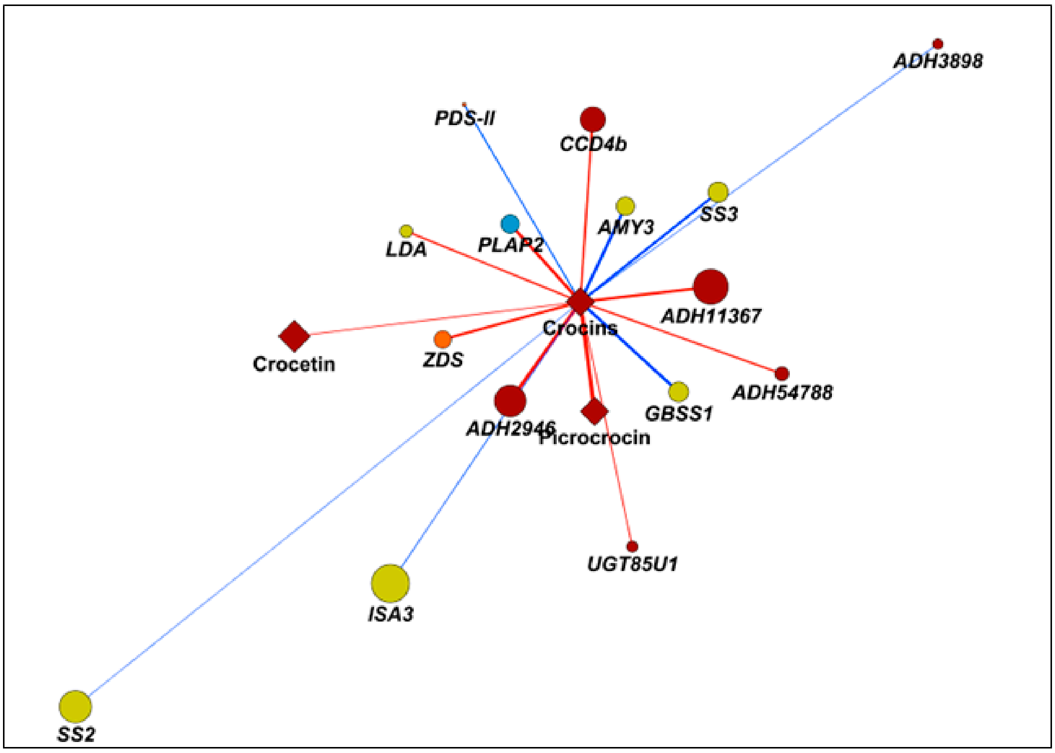

Figure S5. Correlation network of saffron chromoplast-related genes/metabolites using crocins as central hub. Genes and metabolites are represented as rounds and diamonds. Different colors identify genes/metabolites involved in MEP (turquoise), carotenoid (orange), apocarotenoid (burgundy), plastid development (green), and starch (yellow) biosynthesis. Blue and red edges refer, respectively, to negative and positive correlations; only correlations >|0.65| are shown. Edge width and node size were drawn according, respectively, the Pearson correlation coefficient (|p|) and the node strength (ns). Edge length was inversely proportional to |p|.
